# Arterial duct stent versus surgical shunt for patients with duct-dependent pulmonary circulation: a meta-analysis

**DOI:** 10.1186/s12872-020-01817-2

**Published:** 2021-01-06

**Authors:** Dongxu Li, Xu Zhou, Mengsi Li

**Affiliations:** 1 Department of Cardiovascular Surgery, West China Hospital, Sichuan University, No. 37 Guo Xue Xiang, Chengdu, 610041 Sichuan People’s Republic of China; 2 Evidence-Based Medicine Research Center, Jiangxi University of Traditional Chinese Medicine, Nanchang, People’s Republic of China; 3 Department of Anesthesiology, West China Hospital, Sichuan University, Chengdu, People’s Republic of China

**Keywords:** Duct-dependent pulmonary circulation, Palliation, Stent, Shunt

## Abstract

**Background:**

Both systemic-pulmonary shunt and arterial duct stent could be the palliation of duct-dependent pulmonary circulation. We aimed to compare the safety and efficacy of the two approaches.

**Methods:**

The PubMed, EMBASE, and Cochrane Library databases were searched through December 2019 for studies comparing stent implantation and surgical shunt in duct-dependent pulmonary circulation. The baseline characteristics included ventricle physiology and cardiac anomaly. The main outcomes were hospital stay and total mortality. Additional outcomes included procedural complications, intensive care unit (ICU) stay, pulmonary artery growth at follow-up, and other indexes. A random- or fixed-effects model was used to summarize the estimates of the mean difference (MD)/risk ratio (RR) with 95% confidence intervals (CIs).

**Results:**

In total, 757 patients with duct-dependent pulmonary circulation from six studies were included. Pooled estimates of hospital stay (MD, − 4.83; 95% CI − 7.92 to − 1.74; p < 0.05), total mortality (RR 0.44; 95% CI 0.28–0.70; p < 0.05), complications (RR 0.49; 95% CI 0.30–0.81; p < 0.05) and ICU stay (MD, − 4.00; 95% CI − 5.96 to − 2.04; p < 0.05) favored the stent group. Significant differences were found in the proportions of patients with a single ventricle (RR 0.82; 95% CI 0.68–0.98; p < 0.05) or a double ventricle (RR 1.23; 95% CI 1.07–1.41; p < 0.05) between the stent and shunt groups. Additionally, pulmonary artery growth showed no significant differences between the two groups.

**Conclusion:**

Arterial duct stent appears to have not inferior outcomes of procedural complications, mortality, hospital and ICU stay, and pulmonary artery growth in selected patients compared with a surgical shunt.

***Trial registration*:**

CRD42019147672.

## Introduction

In young patients with diminished pulmonary blood flow, a patent ductus arteriosus (PDA) is needed to maintain stable hemodynamics. Such a condition often occurs in neonates with complex congenital heart disease whose hemodynamic stability depends on a PDA. These cardiac lesions are called duct-dependent congenital heart defects [1].

The conventional emergency treatment to maintain pulmonary blood flow is prostaglandin E infusion [2]. However, this treatment can be administered only by intravenous infusion, hence the impracticality of keeping neonates with duct-dependent pulmonary circulation in hospital until the time of definitive surgery [3]. Then, a surgical shunt called the Blalock–Taussig shunt (BTS), which is a direct end-to-side anastomosis of the subclavian artery to the ipsilateral pulmonary artery, is introduced and later modified by the interposition of a tube graft (modified BTS) [4]. Over time, other different surgical systemic-pulmonary artery shunts have been proposed [5]. Despite its widespread use and technical improvements, surgical shunt has been reported to be associated with significant mortality and morbidity [6, 7].

With minimally invasive transcatheter approaches, ductus stent implantation has long been proposed as an effective alternative to surgical systemic-pulmonary artery shunt in patients with duct-dependent pulmonary circulation [8–10]. However, complications such as worsening cyanosis, bleeding, vessel rupture, arterial duct spasm or acute stent thrombosis have also occurred after stenting [11].

Therefore, the safety and efficacy of arterial duct stent and surgically created shunts in patients with duct-dependent pulmonary circulation are still controversial. Consequently, we performed this meta-analysis to compare the outcomes of the two approaches in an attempt to support evidence for clinical strategies.

## Methods

### Search strategy

This study was conducted in accordance with the Preferred Reporting Items for Systematic Reviews and Meta-Analyses guidelines (Additional file 1) and was registered on PROSPERO international prospective registry of systematic reviews (CRD42019147672) [12]. A literature search of computerized medical literature was performed using the PubMed, EMBASE, and Cochrane Library databases. The detailed search strategy was “duct*[tw] AND stent*[tw] AND shunt*[tw]” via PubMed. The search was conducted for published papers from the inception of the databases until December 2019 without language restrictions. To ensure that the search was complete, the reference lists of all retrieved articles were manually searched by the two authors to identify additional relevant studies.

### Inclusion and exclusion criteria

All included studies were required to report the baseline characteristics of patients, and original data for dichotomous and continuous variables were required to be provided or assessable from the data source. Studies were selected using the following inclusion criteria: (1) patients with duct-dependent pulmonary circulation; (2) comparison of treatments including ductus stent implantation and surgically created shunt; and (3) reported at least one of the following outcomes after treatments: (a) length of hospital stay; (b) early or late mortality; or (c) growth of pulmonary arteries at follow-up. Single-arm studies involving only one of the two approaches were excluded. Additionally, letters, editorials, animal trials, case reports, and literature reviews were excluded.

### Study quality and level of evidence

The methodological quality of the included studies was assessed by the two authors. Based on the extracted data, the quality of the included studies was evaluated using the nine-item Newcastle–Ottawa quality scale, a widely used tool to assess quality of nonrandomized trials by a risk evaluation of the adequacy of selection, comparability, and outcome assessment [13]. A high-quality study was defined as a study with a score ≥ 6.

### Data extraction and outcomes of interest

According to a prespecified protocol, all data were extracted independently by the two authors. The following data were extracted from each eligible study using a standardized data collection form: first author’s name, study design, publication year, country where the study was conducted, sample size, age, weight, sex, main diagnosis, ventricle physiology, conduit size, device manufacturer and follow-up interval. The primary outcomes were length of hospital stay (day) and total mortality. Additional outcomes were as follows: a) procedural complications, unplanned reintervention, death in hospital, and intensive care unit (ICU) stay (day) measured in the hospital; and b) diameters of left and right main pulmonary arteries (mm), Nakata index (mm^2^/m^2^), McGoon ratio, oxygen saturation (%), and the time to the next stage or definitive surgical repair (day) measured at follow-up.

### Statistical analysis

The measures of the effects of interest were the mean difference (MD)/risk ratio (RR) with 95% confidence intervals (CIs). We used Cochran’s chi-square test (Q test) and the I^2^ test to evaluate the level of heterogeneity across the studies. If the result of an analysis exhibited a p < 0.05 or I^2^ > 50%, the studies were considered homogeneous, and a random-effects model was used [14]. Otherwise, a fixed-effect model was used for meta-analysis. We explored the source of heterogeneity using sensitivity analysis. Moreover, we planned to construct a funnel plot to detect publication bias across the studies; however, none of the outcomes met the criteria of including a minimum of 10 studies [15]. All statistical analyses were performed using Review Manager software (version 5.3; Cochrane Collaboration, Oxford, UK) and Stata software (version 14.0; Stata Corp., College Station, TX, USA).

## Results

### Search results and characteristics of the included studies

**Fig. 1:**
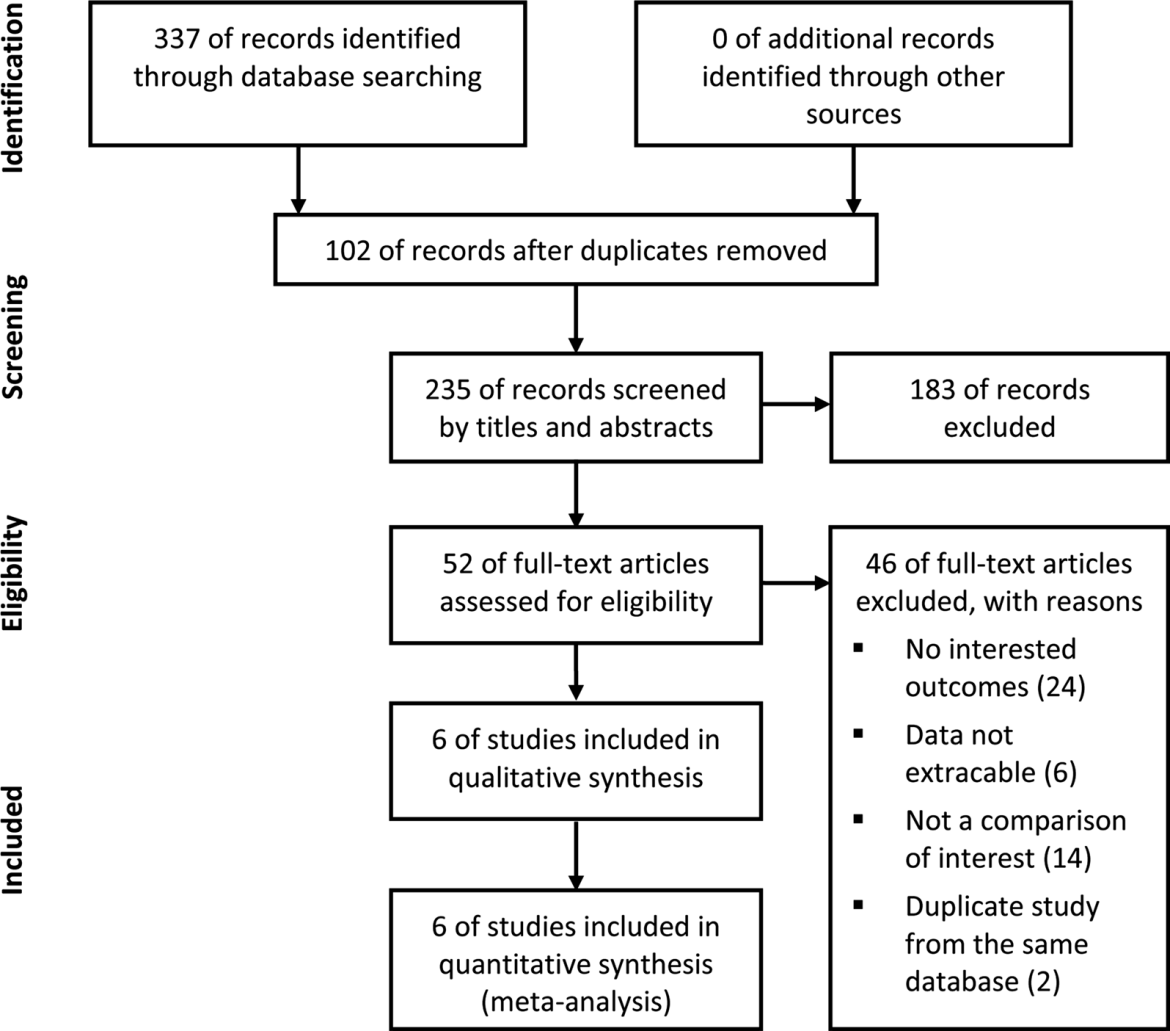
Flow diagram of the literature search and study selection. After the database search, studies were selected and assessed for eligibility, and six studies were finally included in the meta-analysis

According to the inclusion and exclusion criteria, six studies (from 2009 to 2018, involving 757 patients) were included in the analysis [16–21]. Three studies used the same database, and we included only the largest one by Glatz et al. for the synthetic analysis [20, 22, 23]. The detailed literature screen steps are shown in Fig. 1. The baseline characteristics of the patients are presented in Table 1. Most of the included patients underwent initial palliation at less than 30 days of age. The follow-up period until next stage repair was different among the included studies, ranging from 3 months to 8.5 years. According to the Congenital Heart Surgery Nomenclature and Database Project [24], we tried to categorize patients as having either a single or double ventricle. A total of 85 patients had a single ventricle and 130 patients had a double ventricle in the stent group, and 231 patients had a single ventricle and 238 patients had a double ventricle in the shunt group.

**Table 1:** Characteristics of included studies

Study	Year	Region	Design	Group	N	Age (day)*	Weight (kg)*	Ventricle physiology (SV/DV, n)	Cardiac anomaly	Conduit size (mm)*	Device manufacturer	Follow up (year)*
Santoro [16]	2009	Italy	RC	Stent	13	22 ± 39	3.2 ± 0.9	NA	TOF = 2, PAIVS = 6, others = 5	3.3 ± 0.4	Medtronic	0.6 ± 0.3
				Shunt	14	21 ± 30	3.3 ± 1.2	NA	TOF = 6, PAIVS = 2, others = 6	4.4 ± 0.3	Gore-Tex	1 ± 0.5
Amoozgar [17]	2012	Iran	RC	Stent	15	56 ± 60.2	3.9 ± 2.5	4/11	TOF = 2, PA = 10, TA = 2, TGA = 1	3.8 ± 0.3	Boston, Skylor, Blazer, Abbott	0.5
				Shunt	20	102.5 ± 81	5 ± 1.6	5/15	TOF = 4, PA = 9, TA = 3, DORV = 1, others = 3	NA	NA	0.5
McMullan [18]	2014	USA	RC	Stent	13	18.3 ± 15.4	3.3 ± 2.0	2/11	TOF = 1, PA = 8, TGA = 2, others = 2	NA	Abbott	0.5 (0–1)
				Shunt	42	12 ± 54	3.3 ± 1.6	5/37	TOF = 7, DORV = 7, PA = 12, TA = 4, TGA = 8, others = 4	NA	Gore-Tex	0.5 (0–1)
Mallula [19]	2015	USA	RC	Stent	13	7.3 ± 5.3	3.3 ± 0.4	0/9	PAIVS = 13	3.5 ± 0	Boston, Medtronic, Abbott	2 (0.1–6)
				Shunt	16	10 ± 6.5	2.9 ± 0.5	8/5	PAIVS = 16	3.5 ± 0.3	NA	2.5 (0.3–8.5)
Glatz [20]	2018	USA	RC	Stent	106	9 ± 1.7	3.2 ± 0.2	42/64	PAIVS = 47, PAVSD = 18, TA = 5, PS = 36	NA	NA	NA
				Shunt	251	6 ± 1.8	3.1 ± 0.1	139/112	PAIVS = 50, PS = 63, PAVSD = 99, TA = 39	NA	NA	NA
Bentham [21]	2018	UK	RC	Stent	83	8 ± 1.5	3.1 ± 0.1	37/44	TOF = 6, PAIVS = 23, PAVSD = 22, DORV = 6, TA = 6, TGA = 15, others = 5	3.8 ± 0.3	NA	1
				Shunt	171	8 ± 1.7	3.1 ± 0.1	82/74	TOF = 18, PAIVS = 32, PAVSD = 31, DORV = 17, TA = 17, TGA = 24, others = 32	3.6 ± 0.2	Gore-Tex	1

*Kg* kilogram, *mm* millimeter, *yr* year, *RC* retrospective cohorts, *SV* single ventricle, *DV* double ventricle, *TOF* tetralogy of fallot, *PA* pulmonary atresia, *PAIVS* pulmonary atresia with intact ventricular septum, *PSVSD* pulmonary stenosis with ventricular septum defect, *PAVSD* pulmonary atresia with ventricular septum defect, *TGA* transposition of great arteries, *TA* tricuspid atresia, *DORV* double outlets of right ventricle, *NA* not available

^*^Continuous variables are presented as mean ± standard deviation or median (interquartile range)

Four studies were included in the synthetic analysis of the ventricle physiology. We found that the proportion of patients with a single ventricle (RR 0.82; 95% CI 0.68–0.98; p < 0.05) was lower, but the proportion of patients with a double ventricle (RR 1.23; 95% CI 1.07–1.41; p < 0.05) was higher in the stent group than the shunt group (Table 2 and Additional file 2). Additionally, there were differences in the proportions of patients with tetralogy of Fallot or double outlet right ventricle (RR 0.58; 95% CI 0.35–0.96; p < 0.05), or tricuspid atresia (RR 0.50; 95% CI 0.28–0.90; p < 0.05) between two groups.

**Table 2:** The pooled estimates of baseline characteristics and secondary outcomes

Parameters	Studies	Participants	RR/MD	95% CI	p value	Model	Heterogeneity	
							I^2^ (%)	p value
*Baseline characteristics*								
Age	6	757	0.64	− 1.98, 3.26	0.63	Random	96	< 0.001
Weight	6	757	0.07	− 0.03, 0.17	0.18	Random	79	< 0.001
Conduit size	4	345	− 0.44	− 1.72, 0.83	0.50	Random	99	< 0.001
Cardiac anomaly								
PAIVS	4	667	1.67	0.73, 3.85	0.23	Random	95	< 0.001
PAVSD	2	611	0.79	0.18, 3.46	0.75	Random	95	< 0.001
TOF/DORV	4	371	0.58	0.35, 0.96	0.03	Random	0	0.62
TGA	3	344	1.24	0.73, 2.13	0.42	Random	0	0.64
TA	4	701	0.50	0.28, 0.90	0.02	Random	0	0.48
Ventricle physiology								
Single ventricle	4	701	0.82	0.68, 0.98	0.03	Fixed	0	0.50
Double ventricle	4	701	1.23	1.07, 1.41	0.004	Fixed	47	0.13
Perioperative outcomes								
Procedural complications	4	476	0.49	0.30, 0.81	0.005	Fixed	0	0.46
Unplanned reintervention	5	727	1.20	0.56, 2.56	0.64	Random	77	0.005
Early mortality	5	505	0.70	0.28, 1.76	0.44	Fixed	3	0.39
ICU stay	2	611	− 4.00	− 5.96, − 2.04	< 0.001	Random	98	< 0.001
Follow-up outcomes								
Time to repair	5	722	− 23.43	− 57.73, 10.87	0.18	Random	95	< 0.001
Diameter of LPA	2	289	− 0.39	− 0.68, − 0.11	0.006	Random	65	0.09
Diameter of RPA	2	289	0.49	− 0.30, 1.27	0.22	Random	96	< 0.001
Nakata index	4	673	7.32	− 13.89, 28.52	0.50	Random	96	< 0.001
McGoon ratio	2	62	0.10	− 0.13, 0.33	0.39	Fixed	0	1.00
SaO2	5	400	0.23	− 2.37, 2.83	0.86	Random	79	< 0.001

*RR* risk ratio, *MD* mean difference, *CI* confidential interval, *PAIVS* pulmonary atresia with intact ventricular septum, *PAVSD* pulmonary atresia with ventricular septum defect, *TOF* tetralogy of Fallot, *DORV* double outlets of right ventricle, *TGA* transposition of great arteries, *TA* tricuspid atresia, *ICU* intensive care unit, *LPA* left pulmonary artery, *RPA* right pulmonary artery, *SaO2* oxygen saturation

Most reported systemic-pulmonary shunt was BTS or modified BTS, except one study by Mallula et al., which did not report the entailed technique [19]. Three studies were conducted in the United States, one in Iran, one in Italy, and one in the United Kingdom. All included studies were retrospective cohorts, two of which were multicenter studies. According to the Newcastle–Ottawa quality scale [13], six studies were of high quality with a score ≥ 6, respectively (Table 3).

**Table 3:** Quality assessment of included studies with the Newcastle–Ottawa scale

Study	Representativeness of the exposed cohort	Non-exposed cohort drawn from the same community	Ascertainment of exposure	Outcome of interest not present at start	Comparability of cohorts on the basis of design and analysis	Quality of outcome assessment	Follow-up long enough for outcomes to occur	Complete accounting for cohorts	Total score
Santoro [16]	*	*	*	*	*	*	–	*	7
Amoozgar [17]	*	*	*	*	*	*	–	*	7
McMullan [18]	*	*	*	*	*	*	–	*	7
Mallula [19]	*	*	*	*	*	*	*	*	8
Glatz [20]	*	*	*	*	**	*	–	–	7
Bentham [21]	*	*	*	*	**	*	*	*	9

### Primary outcomes

**Fig. 2:**
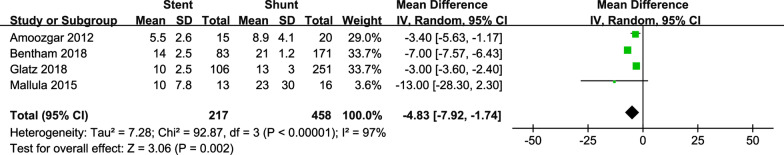
Forest plots of the length of hospital stay. The pooled estimates of hospital stay favored the stent group. *CI* confidence interval

**Fig. 3:**
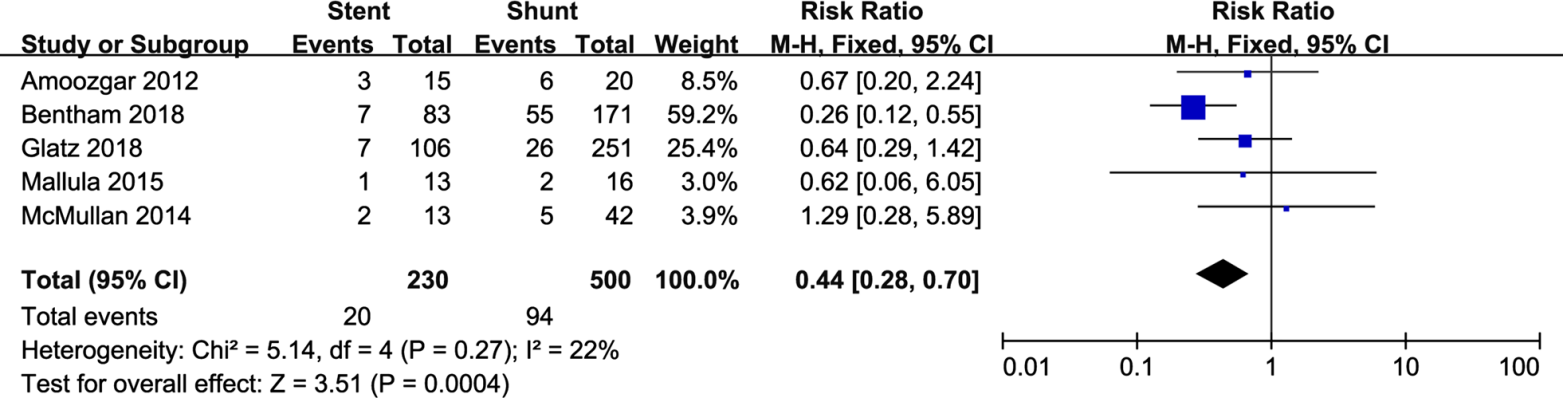
Forest plots of total mortality. The pooled estimates of total mortality favored the stent group. *CI* confidence interval

Four studies compared the length of hospital stay between the stent and shunt groups (Fig. 2). The pooled estimates of hospital stay (MD − 4.83; 95% CI − 7.92 to − 1.74; p < 0.05) favored the stent group. Five studies compared the total mortality between the two groups (Fig. 3). The pooled estimates of total mortality (RR 0.44; 95% CI 0.28–0.70; p < 0.05) also favored the stent group.

### Secondary outcomes

The secondary outcomes are shown in Table 2 and Additional file 2. Synthetic analysis of the some of the secondary outcomes was conducted in only 2 studies. For ICU stay, both the pooled estimates (MD − 4.00; 95% CI − 5.96 to − 2.04; p < 0.05) and individual results of the original studies by Glatz et al. (4 ± 1.5 vs. 7 ± 2; p < 0.05) and Bentham et al. (2 ± 1 vs. 7 ± 1.8; p < 0.05) favored the stent group [20, 21].

Even though the pooled estimates of the diameters of the left pulmonary artery at follow-up (MD − 0.39; 95% CI − 0.68 to − 0.11; p < 0.05) favored the shunt group, the individual results of the original studies by Amoozgar et al. (5.3 ± 0.5 vs. 5.5 ± 0.5; p = 0.23) and Bentham et al. (6.1 ± 0.3 vs. 6.6 ± 0.5; p = 0.47) showed no significant differences between the two groups [17, 21]. Although the pooled estimates of the diameters of the right pulmonary artery at follow-up (MD 0.49; 95% CI − 0.30 to 1.27; p = 0.22) and the individual results of the study by Bentham et al. (7.0 ± 0.4 vs. 6.9 ± 0.4, p = 0.51) showed no significant differences between the two groups, the result of the original study by Amoozgar et al. (5.0 ± 0.5 vs. 4.1 ± 0.5; p < 0.05) favored the stent group [17, 21].

For the McGoon ratio, both the pooled estimates (MD 0.1; 95% CI − 0.13 to 0.33; p = 0.19) and individual results of the studies by Santoro et al. (2.1 ± 0.3 vs. 2.0 ± 0.5, p > 0.05) and Amoozgar et al. (1.9 ± 0.5 vs. 1.8 ± 0.5, p = 0.87) showed no significant differences between the two groups [16, 17].

Other pooled estimates of outcomes (except procedure-related complications (RR 0.49; 95% CI 0.30–0.81; p < 0.05), which favored the stent group) such as unplanned reintervention, early mortality, time to definitive repair, Nakata index, and oxygen saturation at follow-up, showed no significant differences between the stent and shunt groups.

### Sensitivity analysis

**Fig. 4:**
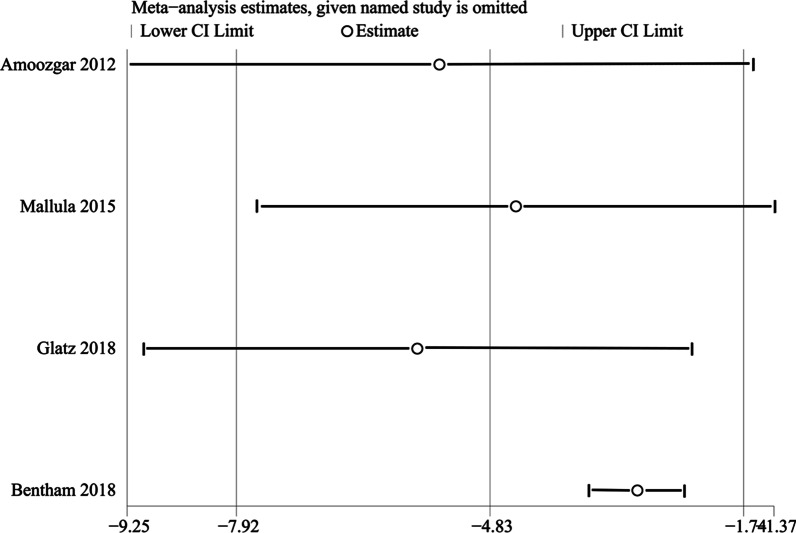
Sensitivity analysis of hospital stay. We found that the study by Bentham et al. had marked effects on the pooled estimates, which meant that the heterogeneity mainly originated from their study

We conducted sensitivity analysis to ascertain the primary origin of the heterogeneity in the pooled estimates of hospital stay. Figure 4 shows that the study by Bentham et al. [21] has marked effects on the pooled estimates. After excluding this study, the pooled estimate of hospital stay (MD − 3.04; 95% CI − 3.62 to − 2.46; p < 0.05) still favored the stent group.

### Publication bias

We constructed funnel plots of hospital stay and total mortality to detect publication bias (Additional file 3). Due to the number of included studies less than 10, we could not confirm whether there was publication bias through the funnel plots. More studies were needed to explore publication bias.

## Discussion

To our knowledge, this is the first meta-analysis to compare ductus stent implantation and surgically created systemic-pulmonary shunt in patients with duct-dependent pulmonary circulation. We found that procedural complications, ICU and hospital stay, and total mortality favored the stent group. The proportion of patients with a single ventricular or double ventricle was significantly different between the two groups. Additionally, other outcomes showed no significant differences between the two groups.

Duct-dependent congenital heart defects involve single or double ventricle physiology with kinds of cardiac anomalies, such as pulmonary atresia with intact ventricular septum or ventricular septal defect, tetralogy of Fallot, pulmonary stenosis, tricuspid atresia, transposition of great vessels, Ebstein anomaly, etc.[25]. Even though not all included studies confirmed that there was a tendency in patient selection [17, 18, 21], the proportion of patients with a single ventricle was higher in the shunt group, and the proportion of patients with a double ventricle was higher in the stent group [16, 20].

Many duct-dependent congenital heart defects either require staged palliation or can be corrected only at a later age. In addition, in the current era, BTS as palliation is a procedure that is almost exclusively performed in the neonatal period or early infancy [26]. However, there are still concerns about the significant morbidity and mortality after BTS [27, 28]. Ductus stent implantation, although not widely accepted, has the advantage of being minimally invasive, which avoids a median sternotomy or lateral thoracotomy and exposure to cardiopulmonary bypass [7]. However, in some cases with bizarre, long and tortuous PDA, it still represents a major technical challenge, which could lead to procedural failure and pulmonary artery distortion; thus, we think it is inappropriate to implant the stent [25, 27].

The procedure-related complications of BTS involve thrombosis, pleural effusion, chylothorax, phrenic and vagal nerve palsy, distortion, and distal pulmonary artery stenosis [5]. In addition, the complications of stent implantation involve thrombosis, embolism, ductal spasm, migration of stent, and branch pulmonary artery stenosis [25, 26]. Amoozgar et al. (0 vs. 30%; p < 0.05) and Mallula et al. (7.7% vs. 37.5%; p < 0.05) reported a lower incidence of complications after the stent implantation than the surgical shunt [17, 19]. However, McMullan et al. (0 vs. 7%; p = 1.0) and Glatz et al. (13.2% vs. 21.5%; p = 0.07) reported no significant differences in the incidence of complications between the two groups [18, 20].

Some cases with complications such as thrombosis embolism, migration of stent, stent stenosis, or branch pulmonary stenosis require unplanned reintervention [1, 29]. In addition, intimal proliferation at the implantation site is almost universal in the first 3–6 months, requiring planned reintervention in the majority of patients [10, 30]. Although stent implantation could potentially aggravate branch pulmonary artery stenosis, with standard initial palliation with a surgical shunt, pulmonary arterioplasty is frequently a part of surgical repair, and postoperative branch pulmonary stenosis requiring reintervention is a common late problem in most pulmonary atresia patients [27].

Amoozgar et al. reported the absence of reintervention in the stent and shunt groups including 35 patients [17]. In addition, the incidence of reintervention was similar (stent vs. shunt: 25% vs. 26%) in both groups by McMullan et al. [18]. Mallula et al. (58.3% vs. 14.3%, p < 0.05) and Bentham et al. (39.8% vs. 24.0%, p < 0.05) reported increased reintervention in the stent group [19, 21]. Only Glatz et al. (11.3% vs. 20.7%, p < 0.05) reported decreased intervention in the stent group [20]. When combined with more frequent planned reintervention in another study, the overall reintervention (48.6% vs. 2.2%, p < 0.05) was more common in the stent group [20, 21].

Due to the minimal invasion and lower complications, patients receiving stent implantation are supposed to recover faster than those receiving surgical shunt. Three studies reported postoperative ventilation time, all with a median time of 1 day in the stent group, which was less than 3 or 4 days in the shunt group [19–21]. Relatively, the length of ICU stay and hospital stay were both shorter according to the studies by Glatz et al. and Bentham et al. [20, 21]. Furthermore, Goldtein et al. compared the costs for hospitalization and the first year of life between the two groups and found that there were lower costs for patients who received stent implantation [22].

With respect to mortality, there were no significant differences in early mortality between the two groups reported by each study [16–19]. However, Bentham et al. reported a reduced risk of total death at follow-up (hazard ratio 0.25; 95% CI 0.07–0.85; p < 0.05) in the stent group when compared with the shunt group [21], and Glatz et al. reported no significant differences in the risk of total death (hazard ratio 0.64; 95% CI 0.28–1.47; p = 0.29) between the two groups [20]. Considering risk factors identified for mortality such as preoperative mechanical ventilation and underlying cardiac anatomy, Glatz et al. and Bentham et al. had constructed propensity-adjusted models with hope to avoid the effects of confounders [20, 21]. Although procedural mortality has significantly declined over time, it is still measurable and has driven the consideration of alternative approaches [28]. With decreased total mortality, ductus stent implantation seems to be a preferable alternative.

Since most patients underwent follow-up cardiac catheterization before surgical repair in less than 1 year, the follow-up period before the next stage repair of most studies is no more than 1 year, as shown in Table 1 [16–18, 21]. Santoro et al. (210 ± 90 vs. 360 + 120; p < 0.05) and Mallula et al. (99 ± 67 vs. 131 ± 57; p < 0.05) reported that patients in the stent group waited for less time to definitive repair than the shunt group [16, 19]. However, McMullan et al. (153 ± 136 vs. 196 ± 91; p = 0.20) and Bentham et al. (231 ± 40 vs. 243 ± 32; p = 0.56) reported there were no significant differences in the interval to next stage repair between the two groups [18, 21]. Only Glatz et al. (178 ± 25 vs. 150 ± 15; p < 0.05) reported less waiting time in the shunt group [20].

Both ductus stent and surgical shunt could maintain blood flow to the lungs, thereby promoting the growth of pulmonary arteries [30]. However, the comparisons of the stent and shunt on the growth of pulmonary arteries remain controversial [5]. During the follow-up, our synthetic results showed that there were no significant differences in the growth of pulmonary arteries, except for the left pulmonary artery, which was larger in the shunt group. This finding might be because the surgical shunt produced both the overgrowth of the contralateral pulmonary artery and a lesser development of the ipsilateral pulmonary artery compared with the percutaneous approach, presumably due to unfavorable graft geometry and flow direction to the pulmonary vascular bed [16]. Thus, blood flow through the ductus stent with an optimal angle enters the pulmonary arteries centrally, with the potential for relatively symmetrical flow to each branch pulmonary artery [20]. Stent implantation might provide a more evenly distributed pulmonary blood flow and promote more balanced growth of the pulmonary arteries [16, 27].

After the sensitivity analysis, we found that the study by Bentham et al. [21] was the source of heterogeneity in the pooled estimates of hospital stay. Additionally, we excluded this study and found there was no directional change in the pooled estimates, which suggested that the result was stable. According to Papachristofi et al., many factors, such as individual patient risk, center, surgeon and anesthetist, could have an effect on the length of hospital stay after cardiac surgery [31]. This finding suggested that these factors might affect the hospital stay in the study by Bentham et al. [21], which made this study a source of heterogeneity.

### Limitations

Several limitations exist in this study. First, the number of studies and the sample size are limited due to the comprehensive procedures performed. The results should be drawn with caution due to the generally limited number of studies, small sample size, and heterogeneity in some analyses. Meanwhile, the synthetic analysis of some of the secondary outcomes was conducted in only 2 studies. According to Cochrane Handbook, even though the meta-analysis could be conducted with more than 2 studies, the limited number of included studies could downgrade the quality of evidence level, especially increasing the risk of publication bias [15]. Hence, we did not only report the pooled estimates of some secondary outcomes but also focused on the individual results of those original studies. Second, all included studies were nonrandomized studies. Observational analyses of this nature fail to fully account for selection bias subtly and inadvertently introduced into the study, which cannot be controlled. Third, since original studies did not compare the outcomes between the stent and shunt among the single ventricle and double ventricle subgroups, nor did they only include patients with a single or double ventricle, we were unable to make the subgroup analysis stratified by ventricle physiology, although it may be a potential source of the heterogeneity. Finally, because some limitations could not be overcome, randomized controlled trials with more data and longer follow-up durations are needed to confirm our findings.

## Conclusion

Arterial duct stent appears to have not inferior outcomes of procedural complications, mortality, hospital and ICU stay, and pulmonary artery growth compared with a surgical shunt. It seems to be a safe and effective alternative to a surgical shunt in selected patients with duct-dependent pulmonary circulation.

## Supplementary Information

**Additional file 1**. The PRISMA checklist.

**Additional file 2**. Forest plots of baseline characteristics and secondary outcomes.

**Additional file 3**. Funnel plots of hospital stay and total mortality.

## Data Availability

The datasets used are available from the corresponding author on reasonable request.

## Abbreviations

PDA: Patent ductus arteriosus
BTS: Blalock–Taussig shunt
ICU: Intensive care unit
MD: Mean difference
RR: Risk ratio
CI: Confidence interval
