# Supplementary material for: Arterial duct stent versus surgical shunt for patients with duct-dependent pulmonary circulation: a meta-analysis
Source: BMC Cardiovasc Disord. 2021 Jan 6;21:9. doi: 10.1186/s12872-020-01817-2 (PMC7789398; doi:10.1186/s12872-020-01817-2)
Supplement: Supplementary file 2 — Additional file 2. Forest plots of baseline characteristics and secondary outcomes. [file 12872_2020_1817_MOESM2_ESM.docx]

**Additional file 2. Forest plots of baseline characteristics and secondary outcomes**

**
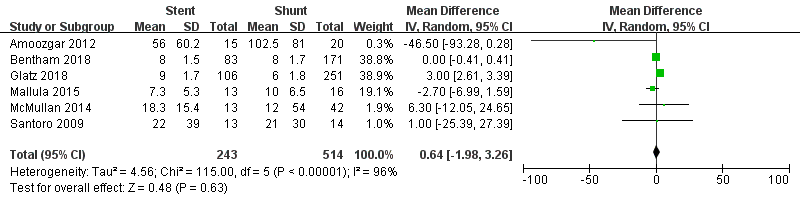
**

**1. Forest plots of age. The pooled estimates showed no differences between stent and shunt groups. CI=confidence interval.**

**
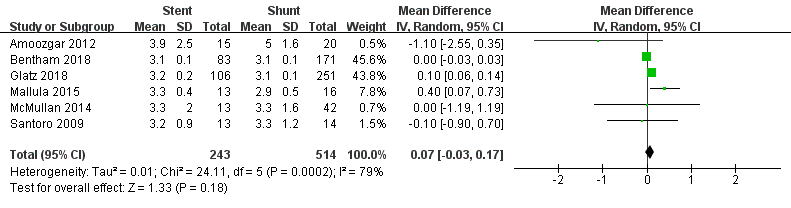
**

**2. Forest plots of weight. The pooled estimates showed no differences between stent and shunt groups. CI=confidence interval.**

**
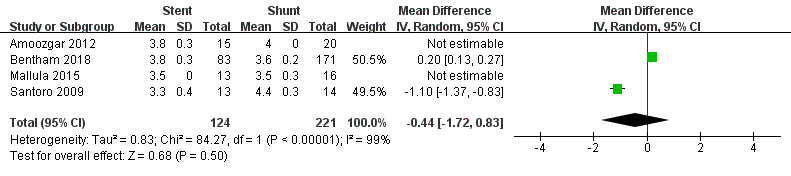
**

**3. Forest plots of conduit size. The pooled estimates showed no differences between stent and shunt groups. CI=confidence interval.**


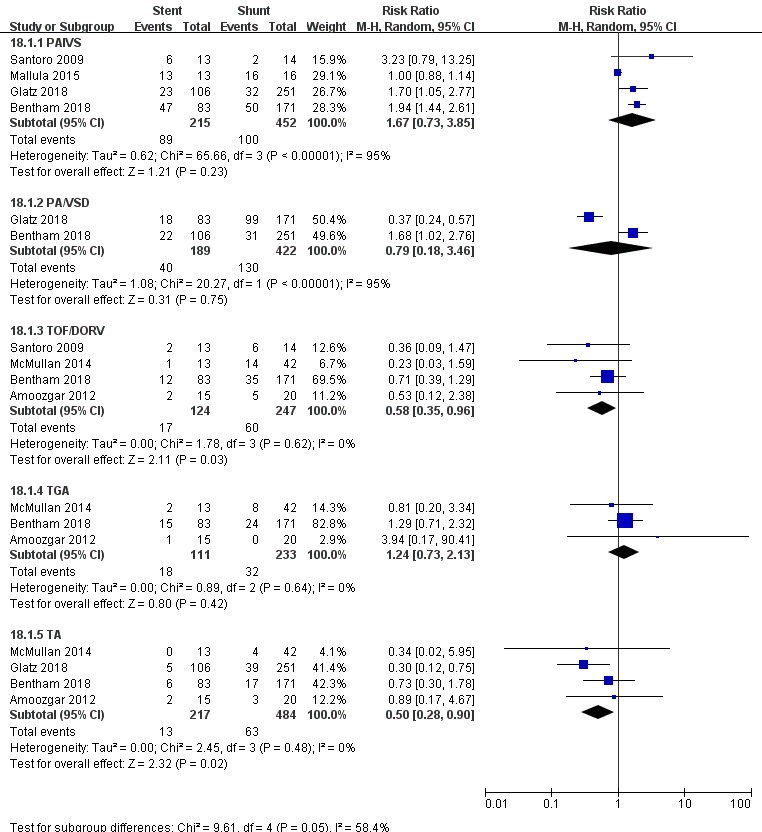


**4. Forest plots of cardiac anomaly. The pooled estimates of subtotals showed no differences between in PAIVS, PAVSD, and TGA between stent and shunt groups; while there were significant differences in TOF/DORV and TA between the two groups. PAIVS, pulmonary atresia with intact ventricular septum; PAVSD, pulmonary atresia with ventricular septum defect; TOF, tetralogy of Fallot; DORV, double outlets of right ventricle; TGA, transposition of great arteries; TA, tricuspid atresia; CI=confidence interval.**


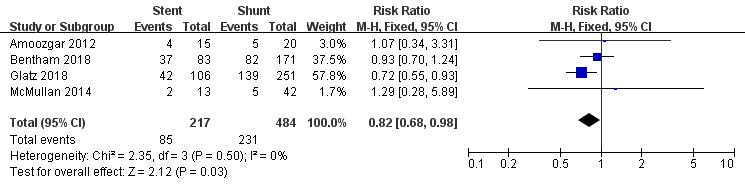


**5. Forest plots of single ventricle physiology. The pooled estimates showed there were more single-ventricle patients in shunt group. CI=confidence interval.**


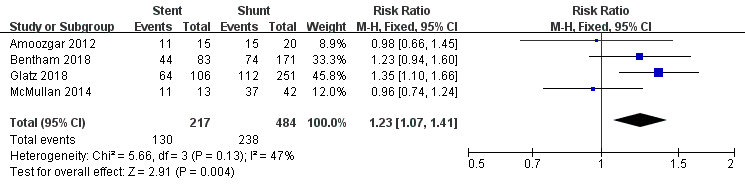


**6. Forest plots of double ventricle physiology. The pooled estimates showed there were more double-ventricle patients in stent group. CI=confidence interval.**


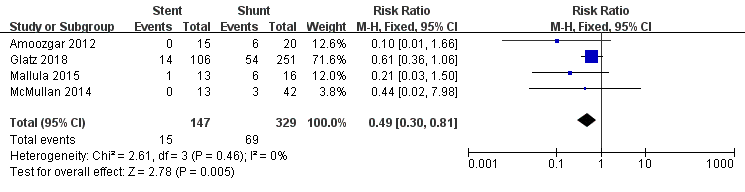


**7. Forest plots of procedural complications. The pooled estimates favored stent group. CI=confidence interval.**


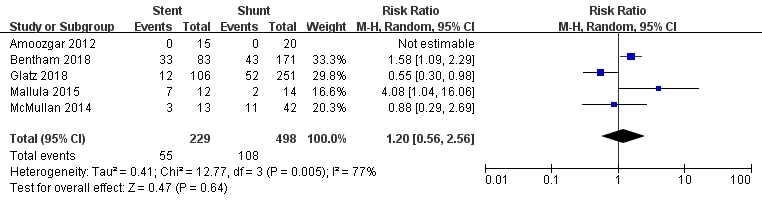


**8. Forest plots of unplanned reintervention. The pooled estimates showed no significant differences between stent and shunt groups. CI=confidence interval.**


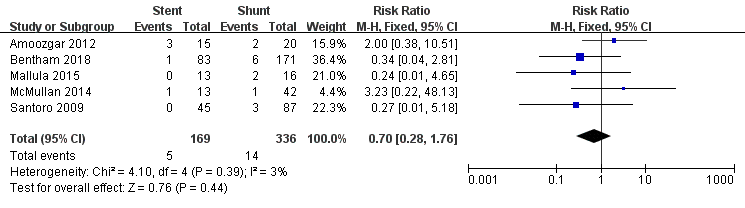


**9. Forest plots of early mortality. The pooled estimates showed no significant differences between stent and shunt groups. CI=confidence interval.**


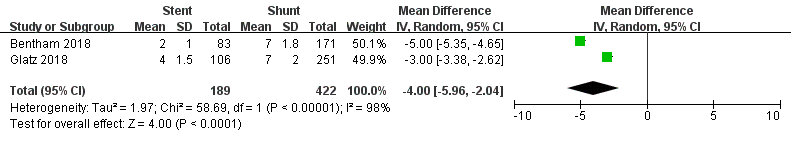


**10. Forest plots of length of ICU stay. The pooled estimates favored stent group. ICU=intensive care unit; CI=confidence interval.**


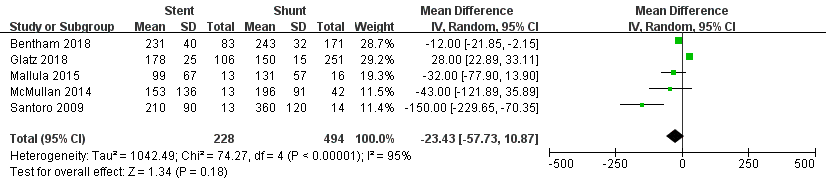


**11. Forest plots of time to definitive surgical repair. The pooled estimates showed no significant differences between stent and shunt groups. CI=confidence interval.**


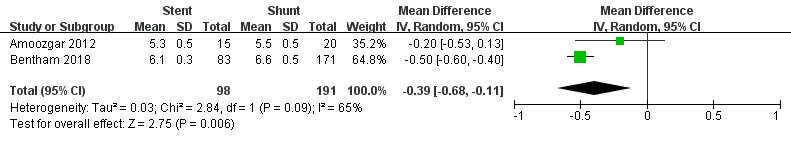


**12. Forest plots of diameter of LPA. The pooled estimates favored shunt group. LPA=left pulmonary artery; CI=confidence interval.**


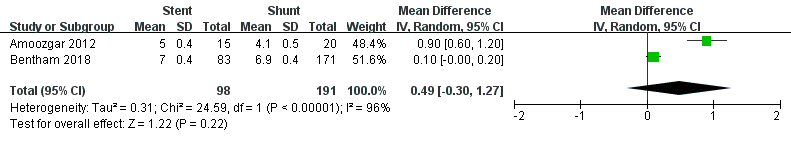


**13. Forest plots of diameter of RPA. The pooled estimates showed no significant differences between stent and shunt groups. RPA=right pulmonary artery; CI=confidence interval.**


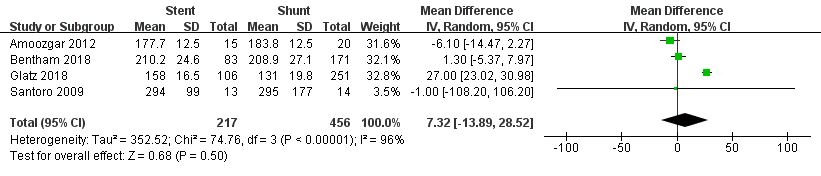


**14. Forest plots of Nakata index. The pooled estimates showed no significant differences between stent and shunt groups. CI=confidence interval.**


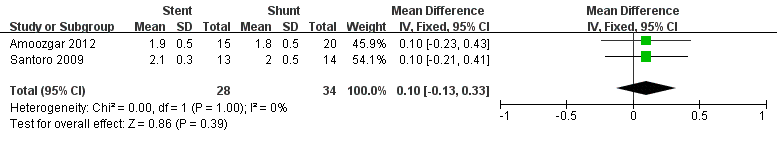


**15. Forest plots of McGoon ratio. The pooled estimates showed no significant differences between stent and shunt groups. CI=confidence interval.**


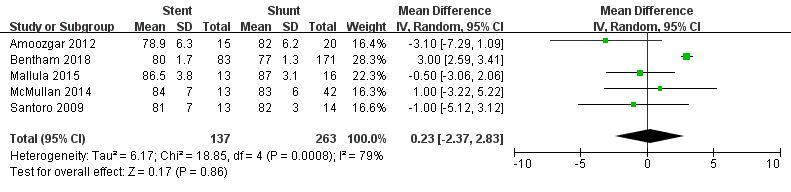


**16.** **Forest plots of SaO2. The pooled estimates showed no significant differences between stent and shunt groups. SaO2=oxygen saturation; CI=confidence interval.**
