# Supplementary material for: Arterial duct stent versus surgical shunt for patients with duct-dependent pulmonary circulation: a meta-analysis
Source: BMC Cardiovasc Disord. 2021 Jan 6;21:9. doi: 10.1186/s12872-020-01817-2 (PMC7789398; doi:10.1186/s12872-020-01817-2)
Supplement: Supplementary file 3 — Additional file 3. Funnel plots of hospital stay and total mortality. [file 12872_2020_1817_MOESM3_ESM.docx]

**Additional file 3. Funnel plots of hospital stay and total mortality.**


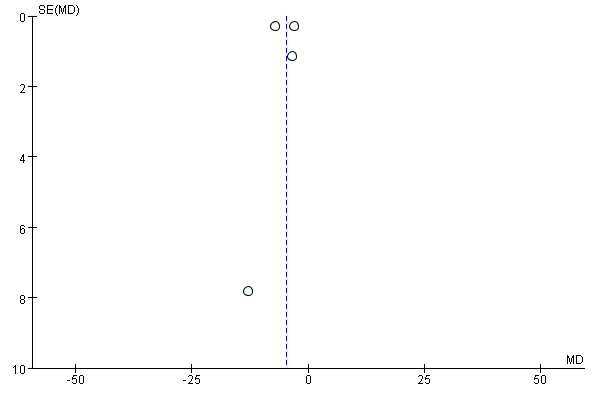


1. **Funnel plots of hospital stay. MD = mean difference, SE = standard error**


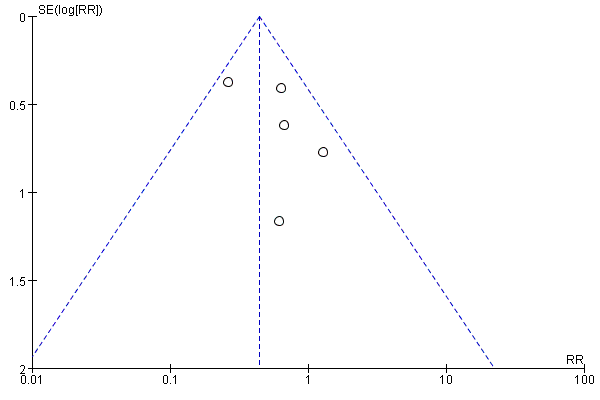


1. **Funnel plots of total mortality. RR = risk ratio, SE = standard error**
